# Parental military deployment as risk factor for children’s mental health: a meta-analytical review

**DOI:** 10.1186/s13034-019-0287-y

**Published:** 2019-06-21

**Authors:** Katrin Cunitz, Claudia Dölitzsch, Markus Kösters, Gerd-Dieter Willmund, Peter Zimmermann, Antje Heike Bühler, Jörg M. Fegert, Ute Ziegenhain, Michael Kölch

**Affiliations:** 1 Department of Child and Adolescent Psychiatry/Psychotherapy, University Hospital of Ulm, Steinhövelstr. 5, 89075 Ulm, Germany; 2 Department of Psychiatry II, Bezirkskrankenhaus Günzburg, Ulm University, Ludwig-Heilmeyer-Str. 2, 89312 Günzburg, Germany; 3 Bundeswehr Hospital Berlin, Center for Psychiatry and Psychotraumatology, German Armed Forces Centre of Military Mental Health, Scharnhorststraße 13, 10115 Berlin, Germany; 4 Department of Child and Adolescent Psychiatry, Rostock University Medical Center, Gehlsheimer Straße 20, 18147 Neuruppin, Germany; 5 Institute for Medical Psychology and Medical Sociology, University Hospital of Goettingen, Waldweg 37A, 37073 Goettingen, Germany

**Keywords:** Military deployment, Child mental health, Meta-analysis

## Abstract

There is evidence that military service increases the risk of psychosocial burden for not only service members but also their spouses and children. This meta-analysis aimed to systematically assess the association between military deployment of (at least one) parent and impact on children’s mental health. For this meta-analytic review, publications were systematically searched and assessed for eligibility based on predefined inclusion criteria (studies between 2001 until 2017 involving children with at least one parent working in military services). Measurements were determined by total problem scores of the children as well as symptoms of anxiety/depression, hyperactivity/inattention, and aggressive behavior. Meta-analyses aggregated the effect sizes in random-effect models and were calculated separately for the relation between parental deployment and civilian/normative data and for the relation between parental deployment and non-deployment. Age of the children was used as moderator variable to explore any potential source of heterogeneity between studies. Parental military deployment was associated with problems in children and adolescents compared to civilian/normative samples. Significant effect sizes reached from small to moderate values; the largest effect sizes were found for overall problems and specifically for anxious/depressive symptoms and aggressive behavior. Within the military group, children of deployed parents showed more problem behavior than children of non-deployed parents, but effect sizes were small. Age of the children had no moderating effect. The results emphasize that children of military members, especially with a deployed parent, should be assessed for emotional and behavioral problems.

## Background

Military personnel who have been deployed in war zones or other unstable regions are at an increased risk for developing mental health disorders, including posttraumatic stress disorder [1]. It is recognized that consequences can extend to family members as well, particularly in children whose parents have been deployed [2, 3]. Before the 1970s, studies that dealt with this matter were rare. The term “military family syndrome” first came into use after the Vietnam War to describe the behavioral and psychosocial problems of children of deployed parents, as well as the effects of deployment on the relationship between the child and the parent remaining at home [4]. The number of studies of this phenomenon began to rise following the Gulf War in 1990–1991, and increased considerably after the terrorist attacks in September 2001 which were followed by military interventions such as Operation Iraqi Freedom (OIF), Operation Enduring Freedom (OEF), and Operation New Dawn (OND).

In the United States, both the number and length of deployments have been increasing over the decades. At present, the length, frequency, and number of deployments are the highest in US history, and the periods between the deployments are the shortest [5]. Chandra et al. [6] found that service members in the U.S. typically are deployed a mean of 2.2 times, for durations of 12 to 15 months. Recent data reveal that approximately 2.4 million service members in the US were available as active duty or ready reserve members in 2015 [8], of whom more than 877,000 were parents of one or more children (80% married to a civilian, 5% married to another member of the military, 15% single). Moreover, the number of individuals involved in military interventions is increasing: between 2001 and 2010, over 2.1 million service members in the US were deployed as part of OIF and/or OEF, with 48% of them serving in Iraq or Afghanistan at least twice [7]. Of these, 44% were parents. In all, 1.75 million children in the US had at least one parent in the military. Not since the Vietnam War have so many US families been affected by military-related family separation, combat injury, and death. As the number of deployments increases and their durations lengthen, the consequences for family systems and children mount up.

The impact of deployment can be particularly hard on children, ranging from the need to take on additional responsibility for younger siblings or household duties to fears for the absent parent’s safety. While some of these effects may have positive aspects, such as promoting the acquisition of new skills and autonomy [9], it is more likely that the negative consequences overweigh the positive. The reduced contact with the deployed parent, concerns about that parent’s safety, and the role confusion brought on by taking on too-early and possibly age-inappropriate family responsibilities can lead to physical and mental overload. There may also be a negative impact on the parenting skills of the remaining parent, who too is dealing with worries about the absent partner while taking on additional household responsibilities and earning a living. Such stressors can result in less family involvement, reduced emotional warmth and responsiveness, controlling or rejecting behaviors, and even hostility [10–13]. Moreover, domestic violence, or child abuse and neglect might occur in those families [3, 14–17].

The above factors might be expected to increase the risk of mental health problems in children of deployed parents. However, the one previous meta-analysis that addressed this issue found only a small association of mental health problems (examining internalizing and externalizing symptoms) with parental deployment [18]. The present meta-analysis describes the findings of the association between deployment of at least one parent and the impact on children’s mental health as assessed by total problems, depression/anxiety, hyperactivity/attention problems, and aggressive behavior, and to additionally assess whether the age of the child had an effect on this association.

To summarize, the aims of this meta-analysis were as follows:

- The first aim was to examine the association between deployment of (at least one) parent and impact on children’s mental health in terms of total problems.
- The second aim was to examine the association between deployment of (at least one) parent and impact on children’s specific symptoms of anxiety/depression, hyperactivity/inattention, and aggressive behavior.
- The third aim was to examine if age of the children has a differentiating effect on results.

## Methods

The review was carried out according to the guidelines specified by the Preferred Reporting Items for Systematic review and Meta-Analysis (PRISMA) protocol [19]. Further information about the current report is available online in the PROSPERO protocol [20]. All meta-analyses were performed using the R Project for Statistical Computing (version 3.4.2) and the software package *metafor* [21].

### Literature search

A body of relevant publications was compiled through a systematic search of the electronic database system of the University of Ulm, which includes 5083 databases such as PubMed, EBSCOhost, Web of Science, and PsycARTICLES. The keywords used were (milit* families OR soldier OR army OR veteran OR deployment) AND (child* OR adolescen* OR family) AND (mental health OR mental illness OR mental disorder OR psychiatric illness OR psychiatric disorder). Moreover, eight websites referring to military projects were included [22–29] to identify studies outside the academic publishing. If applicable, relevant publications that were not captured by the keywords but were cited in a retrieved article were manually searched as well.

Three researchers took part in the search. One, designated the independent reviewer, checked the abstracts of all the identified articles and discarded the vast majority as clearly irrelevant, including non-empirical studies, dissertations, and studies that did not involve children or did not include at least one parent in military service. The other two researchers then reviewed the full texts of the articles that remained for relevance. In cases of disagreement, the independent reviewer acted as a mediator. Discrepancies were resolved through discussion until consensus was reached by at least two of the three reviewers. The articles deemed to be relevant were then further assessed according to the criteria below.

### Inclusion criteria

Articles included in the meta-analysis were restricted to those that reported on families of military service members in the United States, had been published between 2001 and 2017, and involved quantitative measures that were concerned with the relationship between deployment of military parents and the presence of mental health problems in their children. The focus was on instruments that assessed symptoms of anxiety/depression, aggressive behavior, and hyperactivity/inattention. Studies that were concerned with child maltreatment, somatic outcomes (e.g., headache), school/academic variables, coping strategies, attachment, family cohesion, parenting, or familial communication were excluded.

### Control groups

The studies selected for inclusion in the meta-analysis were chosen to compare children of deployed military parents to one of two control conditions: children of civilian parents and children of non-deployed military parents. In the first comparison, deployed military parents included personnel of any branch of the armed forces, both active (full-time occupation in military service) and post-combat (recently returned war veterans), but excluded reserve component personnel. If available, data obtained during pre-deployment (in case of multiple deployments), current deployment, and post-deployment periods were pooled. For the civilian sample, data were obtained from the studies if included (N = 9). Information about the characteristics of the civilian samples were quite rare. Information was either not given or minimized to information that data of the civilian samples were collected as part of statewide surveys (e.g. Healthy Kids/Youth Survey). Only one study described the recruiting process of civilian families from health clinics, obstetrical practices, pediatrics office, or parenting classes and that the civilian sample not differed in level of education, age, or child gender. In other cases, studies compared their military samples with normative data (N = 5). For the remaining studies (N = 13) the authors of this meta-analyses did the comparisons of military connected children with normative data as control. In the second comparison, the deployed sample was defined as children with a parent on active duty in a combat zone (if applicable, data from single and multiple deployments were pooled). While the non-deployed sample consisted of children whose parents were reserve component personnel, military personnel who had been deployed but not sent to a combat zone, or personnel who had returned from a deployment more than 12 months ago.

### Coding of studies (cf. Table 1)

The articles included in the meta-analysis were coded for basic descriptive information (authors, year of publication, study title, sample size, age of the children studied, and type of measurement instruments used) and for whether the deployed military families were being compared to civilian families or to non-deployed military families. The outcome measures were a total score for mental health along with separate scores for the subgroups of anxiety/depression, aggressive behavior, and hyperactivity/inattention. Study characteristics of the included articles are shown in Table 1. The types of informants who provided the mental health data were captured. As the data for the same individual cannot be included in a meta-analysis more than once, in studies where there was more than one informant available for the same sample, such as self-reports and reports by either parent, the report of the parent-at-home was preferred. In case of more than one independent report within a study—for instance, parent-reports for younger children and self-reports for adolescents—all independent reports were analyzed. For the determination of age as a moderator variable, children were categorized into three age groups: early childhood (EC; < 6 years), middle childhood (MC; 6 to < 11 years), and adolescence (AD; 11 to < 18 years). When studies reported results separately by age, effect sizes for each age group were recorded and treated as independent outcomes in the moderator analyses (meta-regression).

**Table 1:** Study characteristics

Author	Year	Age group assessed	Instruments				Effect size
			Total problem score	Anxiety/depression	Aggressive behavior	Hyperactivity/inattention	
Kelley et al. [33]	2001	EC	CBCL	–	–	–	SMD
Ryan-Wenger [34]	2001	MC	RCMAS	RCMAS	–	–	SMD
Ahmadzadeh and Malekian [35]	2004	AD	AGQ	CAS	AGQ	–	SMD
Weber and Weber [36]	2005	AD	BPI	–	–	–	SMD
Chartrand et al. [37]	2008	MC	CBCL	CES-DC	–	–	SMD
Chandra et al. [6]	2008	MC AD	SDQ	Emotional problems (SDQ)	Conduct problems (SDQ)	Hyperactivity/inattention (SDQ)	SMD
Flake et al. [38]	2009	MC	PSC	–	–	Attention issues (PSC)	SMD
Morris and Age [39]	2009	AD	SDQ	Emotional problems (SDQ)	Conduct problems (SDQ)	EATQ–R	SMD
Chandra et al. [40]	2010	AD	SDQ	–	Behavior problems (PBFS)	–	SMD
Gorman et al. [41]	2010	EC	PSY DIAG	Anxiety disorder (PSY DIAG)	Pediatric behavioral disorders (PSY DIAG)	ADHD (PSY DIAG)	log OR
Lester et al. [42]	2010	MC	CBCL	MASC	–	–	SMD
Aranda et al. [43]	2011a	MC	PSC	–	–	Attention issues (PSC)	SMD
Aranda et al. [43]	2011b	AD	Y-PSC	–	–	Attention issues (PSC)	SMD
Herzog et al. [44]	2011	MC	CBCL	–	–	–	SMD
Pfefferbaum et al. [45]	2011	AD	BASC–2	Emotional symptoms (BASC–2)	Behavioral symptoms (BASC–2)	Hyperactivity/attention problems (BASC–2)	SMD
Mansfield et al. [46]	2011	AD	PSY DIAG	Depressive and anxiety disorder (PSY DIAG)	Pediatric behavioral disorders (PSY DIAG)	Impulse control disorder (PSY DIAG)	log OR
Reed et al. [47]	2011	AD	HYS	HYS	–	–	log OR
Wilson et al. [48]	2011	MC	SDQ	–	Conduct problems (SDQ)	Hyperactivity/inattention (SDQ)	SMD
Millegan et al. [49]	2013	AD	PSY HOSP	–	–	–	log OR
Cederbaum et al. [50]	2014	AD	Kessler6	Kessler6	–	–	log OR
Hisle-Gorman et al. [51]	2014	MC	PSY DIAG	–	–	ADHD (PSY DIAG)	log OR
Lucier-Greer et al. [52]	2014	AD	CES-DC	CES-DC	–	–	SMD
Gewirtz et al. [53]	2014	MC	BERS–2	–	–	–	SMD
Wilson et al. [54]	2014	EC MC AD	SDQ	Emotional problems (SDQ)	Conduct problems (SDQ)	Hyperactivity/inattention (SDQ)	SMD
Arnold et al. [55]	2015	AD	CES-DC	CES-DC	–	–	SMD
Mustillo et al. [56]	2016	EC MC	SDQ	Emotional problems (SDQ)	Conduct problems (SDQ)	Hyperactivity/inattention (SDQ)	SMD
Meadows et al. [57, 58]	2016	MC AD	SDQ	Emotional problems (SDQ)	Conduct problems (SDQ)	Hyperactivity/inattention (SDQ)	SMD

ADHD, Attention Deficit Hyperactivity Disorder; AD, Adolescence; EC, early childhood; Log OR, log-transformed odds ratio; MC, middle childhood; SMD, standardized mean difference (= Cohen’s d); PSY DIAG/HOSP, psychiatric diagnoses/hospitalization

Questionnaires: AGQ = Aggression Questionnaire-military [59]; BASC-2 = Behavior Assessment System for Children [60]; BERS-2 = Behavioral and emotional rating scale [61]; BPI = Behavioral Problems Index [62]; CAS = Cattle’s Anxiety Scale-military [63]; CBCL = Child Behavior Checklist, pre-/school age form [64, 65]; CES-DC = Center for Epidemiological Studies-Depression Scale for Children [66]; EATQ-R = Early Adolescent Temperament Questionnaire-Revised [67]; HYS = Healthy Youth Survey (excerpts for depression symptoms; [68]; Kessler6 [69]; MASC = Multidimensional Anxiety Scale for Children [70]; PBFS = Problem Behavior Frequency Scale [71]; PSC = Pediatric Symptom Checklist [72]; RCMAS = Children’s Manifest Anxiety Scale [73]; SDQ = Strength and Difficulties Questionnaire [74]; Y-PSC = Youth Pediatric Symptom Checklist [75]

### Meta-analytic and statistical procedures

In this study, meta-analyses aggregated the effect sizes in random-effect models. Meta-analyses were calculated separately for the comparison of deployed vs. civilian (or normative) data and the comparison of deployed vs. non-deployed data. For each comparison, eight different meta-analyses were implemented to calculate the effect sizes of the comparisons involving the total problem score as well as for the subgroups of anxiety/depression, aggressive behavior, and hyperactivity/inattention. For those studies that provided means and standard deviations, the standard mean difference (SMD; Cohen’s d) was calculated, while for studies that provided the number of specific events in a sample (e.g., prevalence of diagnoses, number of children having specific symptoms with clinical relevance), data were summarized using the Log-Transformed Odds Ratio (log OR). To improve the interpretability of SMD and log OR and to increase the comparability with the earlier meta-analysis by Card and colleagues [18], the effect sizes were converted to the correlation coefficient r [30]. A positive value would indicate that children of deployed parents had more problems than controls, while a negative value would indicate the opposite. As per convention, correlation values around 0.10 were considered to be small effect sizes, values around 0.30 were considered medium, and values around 0.50 were considered large [31]. Statistical heterogeneity of the effects was assessed using I^2^ and tested with a Chi^2^-Test (*Q* statistics). I^2^ values of around 25% (*I*^2^ = 25), 50% (*I*^2^= 50), and 75% (*I*^2^ = 75) were considered to represent low, medium, and high heterogeneity, respectively [32].

## Results

**Fig. 1:**
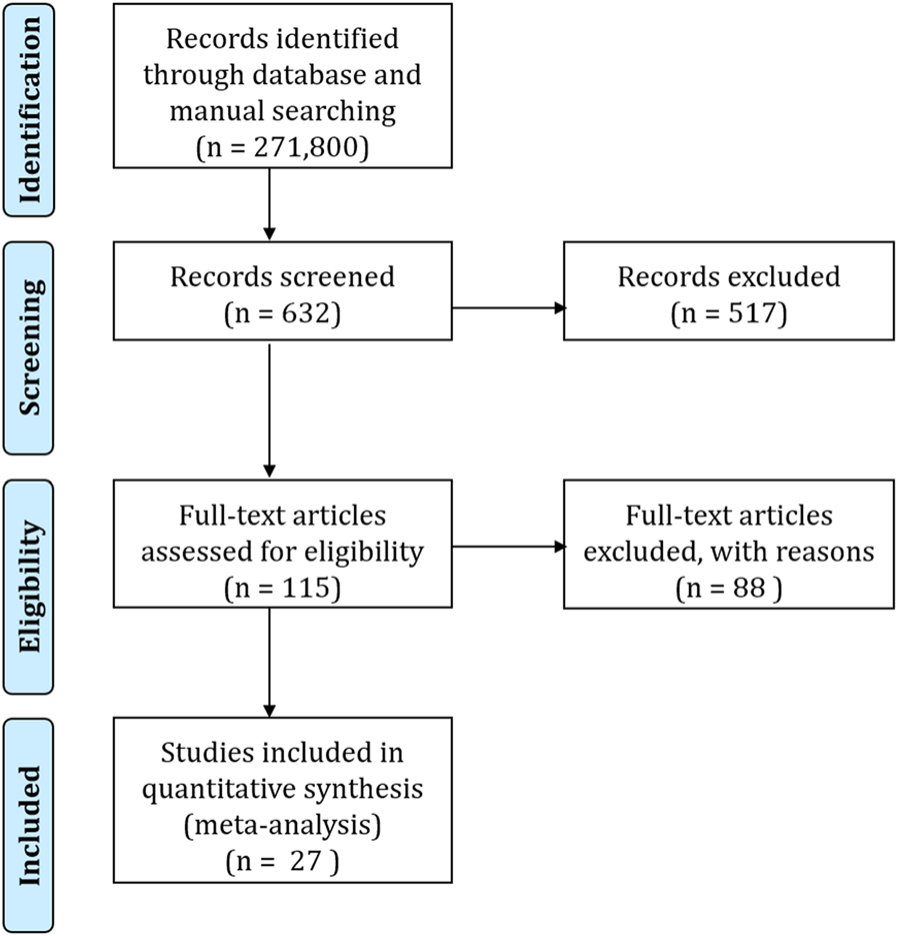
Flow chart of study inclusion process

The initial literature search identified a total of 271,800 articles that contained at least one of the designated key words. The list was reduced to 115 articles after screening for relevance (i.e., the review of abstracts in the first round and review of full text in the second round; see Fig. 1), and was further reduced to 27 after the elimination of studies that did not meet all the inclusion criteria.

As shown in Table 1, the most common instruments used for assessing children’s mental health problems were the Child Behavior Checklist (CBCL; [65]) and the Strength and Difficulties Questionnaire (SDQ; [74]). If a study did not use an instrument that included a score for overall problem behaviors, the total problem score was based on the score for whatever specific problem was being measured (e.g., a questionnaire for anxiety only). Scores for the three symptom subgroups were obtained from whatever instrument was administered; e.g., the Children’s Manifest Anxiety Scale for anxiety, or the emotional problem subscale of the SDQ for anxiety/depression.

Some studies reported results separately for boys and girls or for different age groups; in these cases, the data were averaged before the effect sizes were calculated. Because of the small number of studies, for both comparisons of interest, meta-regression of age was limited to studies that measured the standard mean difference of the total problem score. Several important characteristics, such as stage of deployment, number and length of deployments, nature of deployment, and gender, were frequently omitted in the studies so could not be included as potential moderator variables in the meta-analysis. It must also be noted that the operationalization of “deployment” and “non-deployment” varied across studies, with definitions of the former ranging from deployments that were ongoing during the time of assessment to ones that had ended several weeks earlier, and the latter ranging from only partial participation over an entire military career (e.g., reserve component personnel) to deployments that had ended more than 12 months before the assessments.

The findings of the meta-analyses are shown in Table 2, presented according to whether the effect size was calculated using the standard mean difference (SMD) or Log-Transformed Odds Ratio (log OR). For the military vs. civilian comparison, the analyses included 27 independent samples comprising a total of 880,601 children of military families and 384,432 children of civilian families; for the deployed vs. non–deployed comparison, they included 18 independent samples comprising 341,769 children of deployed parents and 420,264 children of non-deployed parents. Overall, the sample sizes for the individual analyses ranged from 768 to 1,249,100.

**Table 2:** Summary of study outcomes included in meta-analyses

Outcome	k	N	N	Effect size		Confidence interval 95%	Correlation coefficient r	Heterogenity I^2^ (%)
		Military sample	Civilian sample/normative data					
Total problem score	21	6560	103,060	*SMD*	0.51*	0.31–0.70	0.25	98*
	6	874,041	281,372	*log OR*	1.02	-0.63–2.66	0.27	100*
Anxiety/depression	13	4453	54,506	*SMD*	0.55*	0.39–0.70	0.26	94*
	4	339,363	21,798	*log OR*	1.56*	0.45–2.68	0.40	97*
Aggression	9	3582	45,819	*SMD*	0.44*	0.16–0.72	0.21	97*
	2	156,497	6084	*log OR*	1.66	− 0.27–3.59	0.42	100*
Hyperactivity/inattention	10	2085	63,081	*SMD*	0.32*	0.04–0.60	0.16	96*
	3	1,249,100	9126	*log OR*	− 1.22	− 3.88–1.45	0.32	100*

Outcome	k	N	N	Effect size		Confidence interval 95%	Correlation coefficient r	Heterogenity I^2^ (%)
		Deployed sample	Non-deployed sample					
Total problem score	13	1370	1681	*SMD*	0.30*	0.15–0.45	0.15	67*
	5	340,399	418,583	*log OR*	1.37	− 0.82–3.56	0.35	100*
Anxiety/depression	8	1013	1426	*SMD*	0.15*	0.00–0.30	0.08	47
	5	340,419	418,584	*log OR*	0.97	− 0.11–2.05	0.26	100*
Aggression	5	768	911	*SMD*	0.05	− 0.04–0.15	0.03	0.00
	3	335,216	414,236	*log OR*	0.98*	0.07–1.90	0.26	100*
Hyperactivity/inattention	6	818	961	*SMD*	0.08	− 0.10–0.25	0.04	45
	2	193,545	113,185	*log OR*	0.44	− 0.23–1.11	0.12	76*

k, number of studies; N, number of participants

* p < .05

### Military vs. civilian comparison

For the total problem score, data were obtained from all 27 studies. The effect size was significant for the 21 studies in which it was calculated using SMD (0.51*, 95%-CI 0.31–0.70), but was not significant for the six studies calculating the log OR (1.02, 95%-CI − 0.63–2.66). Meta–regression found no significant difference between the three age groups in total problem score (Q_2_ = 2.61, p = 0.27). For the symptom subgroups of anxiety/depression, aggression, and hyperactivity/inattention, information was available from 17, 11, and 13 studies, respectively (for the comparisons on subgroups, see Table 2). Heterogeneity was high for all comparisons, ranging from 94% to 100%.

### Deployed vs. non-deployed comparison

The results were less consistent for these data than for those involving the comparison with civilian families. Here, information was available from 18 studies for the total problem score and from 13, 8, and 8 studies, respectively, for the three subgroup scores. For the total problem score, the effect size calculated using SMD was significant (0.30*, 95%-CI 0.15–0.45) but was smaller than that seen for the comparison with civilian data. The effect size that was calculated using log OR was not significant (1.37, 95%-CI − 0.82–3.56). Meta-regression showed again that age was not a significant moderator, assessed in 18 studies (SMD) for the total problem score (Q2 = 0.40, p = 0.82). For the symptom subgroups, see Table 2 for comparisons. There was again a wide range of heterogeneity, from 0% to 100%.

## Discussion

The aims of this meta-analytic review were to examine the association between deployment of military parents and the impact on the mental health of their children, and to assess the influence of children’s age on this association. The findings indicated that children of deployed parents have higher rates of mental health problems compared to civilian or normative samples as assessed by several measures. Significant differences were seen on some of the comparisons, with effect sizes that reached values ranging from small to moderate. The largest effect sizes were found for the internalizing symptoms of anxiety and depression, which would arise from the existence of fears for the deployed parent’s safety. There is also a possibility that the burdens and worries of the remaining parent are somehow transmitted to children, whether in actual words or via non-verbal indications [76–78]. Children have reported that following deployment of one parent, the other parent shows increases in depression, anger, and stress [79].

An impact of deployment was also seen in the within-group comparison involving military families, with children of deployed parents exhibiting higher rates of both internalizing (anxiety/depression) and externalizing (aggressive behavior) symptoms, as well as higher rates of total problems, compared to children whose parents were not deployed. Since deployment is associated with imminent danger of injury or even death, these symptoms likely are due to greater worries; that is, the negative behavioral consequences are more pronounced in children whose parents are facing greater danger. However, the effect sizes were small, indicating lesser differences than those seen between children of military families and children of civilian families.

The results of this meta-analysis differed from those of Card and colleagues [18], who had found only a small association between parental deployment and mental health problems in children. One possible explanation for the discrepancy is the different time periods covered: Card and colleagues had included nine studies published up to 2001 and seven published afterwards, while all 27 studies included in the current meta-analysis were published between 2001 and 2017. As 2001 was the year of the 9/11 terror attacks which led to several major military interventions (OIF, OEF, and OND), both the number and length of deployments in the US have increased since the time of the meta-analysis by Card and colleagues, and hence the impact of parental deployments on children’s mental health may have been notably increased. In addition, the greater number of studies in the current review (27 vs. 16) may account for some of the difference between the two analyses, as effects are more likely to be detected when more studies are included.

No effect of children’s age was found on mental health status. We had expected the results to be age-dependent, with younger children displaying more problems than older ones, externalizing symptoms in particular. However, in the majority of studies, the samples of children studied were in the categories of middle childhood (6 to < 11 years) and adolescence (11 to < 18 years), with only four studies including samples in the category of early childhood (< 6 years). This unequal distribution might have contributed to the absence of an age effect.

### Limitations

The most significant limitation of this meta-analysis was the heterogeneity of the studies analyzed, i.e., the between-studies variability. According to Higgins and colleagues [24], I^2^ values of 25%, 50%, and 75% can be tentatively classified as low, medium, and high, and several of the values seen here were more than 90%. There were several reasons for the high heterogeneity: the studies used different questionnaires and instruments to evaluate psychopathological symptoms and diagnoses; military members belonged to different branches and ranks within the armed forces; and the status of both deployment and non-deployment was defined in multiple ways. Additionally, some of the comparisons were done using civilian samples and others using normative data, and the civilian samples that were recruited for the analyzed studies might not have been as representative in terms of geographical and educational characteristics as the samples that had been recruited for the normative studies.

Another limitation was that apart from age of the children (which, as described above, was distributed very unequally), no mediators or moderators that might have influenced the findings could be explored, because reporting of the data was too fragmentary to allow for meaningful analyses. Moreover, most of the analyzed studies had cross-sectional designs, so it was not possible to draw conclusions on time-dependent courses or causality. Finally, as the number of studies that met the criteria for being included in the meta-analysis was small (21 studies in the SMD analyses and 6 in the log OR analyses), the statistical power for detecting group differences was limited.

A key component of a well-conducted systematic review is an objective and sensitive literature search of multiple sources. An additional research strategy including the term “parent” in our search criteria did not reveal relevant studies. Moreover, we have undertaken an additional review of appropriate projects of the Department of Defense or of RAND Corporation in the United States to examine potential studies that were partly outside the academic publishing. Most of the projects, such as “Military Family Life Project” [80] and “Blue Star Families, Military Family Lifestyle Survey” [81] did not reach scientific inclusion criteria due to the use of standardized and comparable instruments. Only one study was included, “The Deployment Life Study” [57]. A further promising project is “The Millennium Cohort Family Study”, recently published in December 2018 [82], may include in future reviews.

## Conclusions

Parental military deployment was found to have a negative impact on children’s mental health as indicated by assessment of several psychopathological symptoms. Furthermore, the results suggest that within the military group children of deployed parents showed more problem behavior than children of non-deployed parents. The age of the children was not found to play a role. The fact that a stronger effect was found in this meta-analysis than in an earlier one that had mainly looked at studies conducted prior to the 9/11 terrorist attacks suggests that the impact of parental deployments on children’s mental health has increased significantly since 2001.

The increased risk to children whose parents are in the military needs to be addressed by the health care system as well as through preventive approaches. The results of this meta-analysis stress the continuous need for awareness, especially with regard to internalizing symptoms, of how children in this situation are coping in everyday life, in both family and school settings. In the United States, several interventions have been developed of which some have been positively evaluated; for example, the “Families Overcoming Under Stress (FOCUS)” project [83, 84].

The findings presented here are restricted to the US population, but it is likely that children of military members in other nations carry similar burdens of psychiatric symptoms. With regard to transferability of prevention and intervention programs to other parts of the world, it is important to consider the possible limitations, since such programs depend on national health care and welfare systems which differ from country to country [85]. However, regardless of national differences, all countries with armed forces that are involved in deployment or combat need to ensure the provision of screening measures and preventative interventions that are directed at this vulnerable group.

## Availability of data and materials

The data analyzed during the current study are available from the corresponding author on reasonable request. Further information about the current report is available online in the PROSPERO protocol (ID: 75425; [20]).
